# Atorvastatin Calcium Inhibits Phenotypic Modulation of PDGF-BB-Induced VSMCs via Down-Regulation the Akt Signaling Pathway

**DOI:** 10.1371/journal.pone.0122577

**Published:** 2015-04-15

**Authors:** Shuang Chen, Baoqin Liu, Dehui Kong, Si Li, Chao Li, Huaqin Wang, Yingxian Sun

**Affiliations:** 1 Department of Cardiology, the First Hospital of China Medical University, Shenyang, Liaoning, China; 2 Department of Biochemistry and Molecular Biology, China Medical University, Shenyang Liaoning, China; University of Sassari, ITALY

## Abstract

Plasticity of vascular smooth muscle cells (VSMCs) plays a central role in the onset and progression of proliferative vascular diseases. In adult tissue, VSMCs exist in a physiological contractile-quiescent phenotype, which is defined by lack of the ability of proliferation and migration, while high expression of contractile marker proteins. After injury to the vessel, VSMC shifts from a contractile phenotype to a pathological synthetic phenotype, associated with increased proliferation, migration and matrix secretion. It has been demonstrated that PDGF-BB is a critical mediator of VSMCs phenotypic switch. Atorvastatin calcium, a selective inhibitor of 3-hydroxy-3-methyl-glutaryl l coenzyme A (HMG-CoA) reductase, exhibits various protective effects against VSMCs. In this study, we investigated the effects of atorvastatin calcium on phenotype modulation of PDGF-BB-induced VSMCs and the related intracellular signal transduction pathways. Treatment of VSMCs with atorvastatin calcium showed dose-dependent inhibition of PDGF-BB-induced proliferation. Atorvastatin calcium co-treatment inhibited the phenotype modulation and cytoskeleton rearrangements and improved the expression of contractile phenotype marker proteins such as α-SM actin, SM22α and calponin in comparison with PDGF-BB alone stimulated VSMCs. Although Akt phosphorylation was strongly elicited by PDGF-BB, Akt activation was attenuated when PDGF-BB was co-administrated with atorvastatin calcium. In conclusion, atorvastatin calcium inhibits phenotype modulation of PDGF-BB-induced VSMCs and activation of the Akt signaling pathway, indicating that Akt might play a vital role in the modulation of phenotype.

## Introduction

Vascular smooth muscle cells (VSMCs) are highly specialized cells whose principal function is contraction and regulation of blood vessel tone, thus control of blood pressure and blood flow[1]. It is well known that VSMCs, unlike either skeletal muscle cells or cardiomyocytes, are not terminally differentiated and possess remarkable phenotypic plasticity that permits rapid adaptation to certain environmental cues[2, 3]. In the media layer of mature blood vessels, VSMCs exhibit differentiated and contractile phenotype, typically proliferate at an extremely low rate and have a very low synthetic activity. They express a unique repertoire of contractile markers specific to smooth muscle, such as smooth muscle alpha actin (αSMA), SM22α, smooth muscle myosin heavy chain, calponin and alpha-tropomysin[1]. However, VSMCs can reversibly switch to a dedifferentiated-synthetic state in response to injury such as after angioplasty, stenting, or bypass surgery[4]. This phenotypic modulation is characterized by an increased rate of proliferation, migration, and extracellular matrix protein deposition which contributes to intimal hyperplasia[5–7]. At the same time, they demonstrate low expression of SM-specific contractile markers[3, 8]. This phenotypic switch from contractile phenotype (differentiated state) to synthetic phenotype (dedifferentiated state) acts as a critical factor in different cardiovascular diseases such as atherosclerosis, restenosis after angioplasty or bypass, and hypertension[9, 10]. Although much is reported regarding factors and mechanisms that may control VSMCs phenotype modulation, our current knowledge of the mechanisms controlling VSMCs phenotype switching is far from complete.

It has been well established that multiple cytokines and growth factors are released to stimulate VSMCs proliferation during the repair of vascular injury[11, 12]. For example, the increased production of PDGF-BB stimulates VSMCs proliferation in response to vascular injury via initiating related signaling pathways[13, 14]. Platelet-derived growth factor-BB (PDGF-BB) functions as one of the most potent mitogens and chemoattractants for VSMCs. It has also been demonstrated to stimulate phenotype modulation of VSMCs from differentiated phenotype to dedifferentiated phenotype[7, 15–17]. PDGF-BB binds to PDGF receptor (PDGFR)-B and subsequently activates several intracellular signaling cascades in VSMCs, including phosphatidylinositol 3-kinase (PI3K)/AKT, extracellular signal-regulated kinase (ERK) and p38 mitogen-activated protein kinase (MAPK) pathways[18–20]. Many studies have reported that (PI3K)/AKT signaling pathway is implicated in the PDGF-BB-induced proliferation, migration and the changes of cytoskeleton of VSMCs[21, 22], which are fundamental features involved in the phenotype modulation[23]. Moreover, PDGF-BB not only stimulates proliferation and migration in VSMCs but also changes several genes expression. Previous studies have indicated that PDGF-BB markedly inhibits the expression of multiple VSMCs differentiation markers, including αSMA, calponin and SM22α in cultured VSMCs[24–26].

The lipid-lowering effects of 3-hydroxy-3-methylglutaryl coenzyme A (HMG-CoA) reductase inhibitors (statins), such as atorvastatin calcium (ATV), have been widely established in clinical patients. It has been reported that statins have protective effects against VSMCs proliferation and migration in cardiovascular remodeling[27]. Moreover, accumulating evidence has shown that statins exhibit stabilizing effects on vulnerable atherosclerotic plaques [28] and protect VSMCs from TGF-β1-stimulated calcification[29]. Several studies have shown that atorvastatin may slow atherosclerosis progression and improve the outcomes of coronary heart disease[30]. However, the molecular mechanisms underlying the action of atorvastatin calcium on VSMCs have not been fully elucidated.

Therefore, this study aimed to investigate whether atorvastatin calcium was able to inhibit PDGF-BB-stimulated VSMCs proliferation, migration and phenotype modulation, as well as the associated molecular mechanisms.

## Materials and Methods

### Ethics Statement

All experimental protocols were reviewed and approved by the Ethics Committee of China Medical University (Shenyang, China). All procedures were performed in accordance with the ethical standards. Male Sprague-Dawley rats, 8 weeks, weighing 150–200 g were performed under general anesthesia with pentobarbital sodium (50mg/kg), and all efforts were made to minimize suffering.

### Materials

Atorvastatin calcium was obtained from JiaLin Company (Beijing, China). Recombinant human PDGF-BB was purchased from PeproTech Company (Rocky Hill, NJ 08553, USA). MTT and DMSO were obtained from Sigma-Aldrich (St. Louis, MO, USA). Click-iT^®^ EdU Imaging Kits and Alexa 546-conjugated rhodamine phalloidin were purchased from Invitrogen (Carlsbad, CA, USA). Antibodies for αSMA, SM22α, calponin, phospho-Akt (Ser473), Akt, and glyceraldehyde 3-phosphate dehydrogenase (GAPDH) were purchased from Abcam (USA).

### Isolation and culture of VSMCs

Rat aortic SMCs were isolated from thoracic aortas of male Sprague-Dawley rats by using the collagenase digestion method, and cultured in DMEM (Thermo Fisher, Shanghai, China) medium containing 10% fetal bovine serum (FBS) (Thermo Fisher, Shanghai, China), 100 U/mL penicillin and 100 mg/mL streptomycin at 37.0°C in a humidified atmosphere of 5% CO2. The cultured VSMCs were confirmed by expression of known marker protein smooth muscle α-actin through immunofluorescence assay (S1 Fig). For all experiments, primary rat aortic VSMCs were subcultured and used between passages 4 to 7. VSMCs were grown to 70% to 80% confluence and then made quiescent by incubation in DMEM without FBS for 24 hours before experiments.

### Cell Stimulation by PDGF-BB and Treatment of atorvastatin calcium

VSMCs were grown to 70–80% confluence and pre-cultured in serum-free medium for 24h before treatment. Atorvastatin calcium was dissolved in methanol for a stock solution of 100 mM and then diluted to desired concentrations with media prior to cell treatment. Cells were treated with various concentrations of atorvastatin calcium from 1 to 50 μM in cell proliferation assay, 10 μM in migration assay, cell morphology and western blotting on quiescent cells with or without 20ng/mL PDGF-BB for designated times.

### Cell Proliferation and DNA Synthesis

#### MTT assay

VSMCs were cultured to 80% confluence and serum-free for 24 hours. Cell viability was examined by MTT assay. Cells were seeded in 96-well culture plates at a density of 5000/well and incubated with 0.5mg/mL MTT in the last 4h of the culture period at 37C. Thereafter, the medium was replaced with 100 μl DMSO and the plate was gently rotated on a linear and orbital shaker for 5 min to completely dissolve the precipitation. An automatic microplate reader microplate reader (Bio-Rad, Hercules, CA, USA) was used to determine the absorbance at 570 nm.

#### Edu incorporation assay

DNA synthesis was performed by a 5-ethynyl-2´-deoxyuridine (Edu) incorporation assay (Click-iT^®^ EdU Imaging Kits, invitrogen, USA) according to the manufacturer’s instructions. Briefly, cells were incubated with Edu-labeling solution for 2h at 37°, and then the cells were fixed with 4% cold formaldehyde for 30 min at room temperature. After permeabilization with 1% Triton X-100, the cells were reacted with Click-iT^®^ reaction cocktails (invitrogen) for 30 min. Subsequently, the DNA contents of the cells were stained with Hoechst 33342 for 30 min. Finally, Edu-labeled cells were counted using fluorescence microscopy (CKX41-F32FL, Olympus) and normalized to the total number of Hoechst-stained cells.

### RTCA xCelligence migration assays

The RTCA xCelligence system (Roche Applied Sciences, Almere, the Netherlands), based on cell–electrode substrate impedance detection technology, was used for real-time migration assays. We used CIM plate (Cat# 05665817001, Roche) for migration assay. Briefly, we seeded 4 × 10^4^ cells in serum-free media into each chamber. After all chambers were set up, the CIM plate was put into xCelligence instrument at 37°C, 5% CO2 incubator and cell index was recorded every 15 min intervals. Experiments were performed in triplicate.

### Cell morphology and actin filaments analysis by laser confocal microscopy

VSMCs were cultured in 24-well culture plates. After treatment, VSMCs were fixed with 4% cold formaldehyde for 30 min at room temperature, and then permeabilized with 1% Triton X-100 for 20 min. After being washed with PBS, the cells were incubated with Alexa 546-conjugated rhodamine phalloidin (5U/mL, 1:100, Invitrogen, Carlsbad, CA, USA) for 1 h in the dark. Thereafter, the nuclei were stained with DAPI (Sigma) for 5 min in the dark. Finally, cell morphology and actin filaments were visualized and captured using a fluorescence microscope (Olympus). An area of 100 cells for each group was analyzed by Image J 1.47 software (NIH, Bethesda, MD, USA). The circularity was a function of Image J software and was presented from 0 to 1, with values closer to 0 indicating spindle morphology and those closer to 1 indicating a circular phenotype. We used circularity of Image J software to determine the morphological distribution between the contractile phenotype and the synthetic phenotype of VSMCs. The average circularity ± SEM was obtained from 100 single cells per each type from images of fluorescence microscope and auto-calculated by Image J software.

### Western blot

VSMCs were lysed in cold radio-immunoprecipitation assay (RIPA) lysis buffer (Santa Cruz Biotechnology). Proteins were separated by NuPAGE^®^ Novex 4–12% Bis-Tris Gel (invitrogen) and electrophoresed in the XCell SureLock™ Mini-Cell (invitrogen). Next, proteins were transferred onto a PVDF membrane, blocked, and incubated with primary antibodies at 1: 1000 dilutions. After incubation with secondary antibodies, proteins were detected by enhanced chemiluminescence (ECL) (Amersham, Sunny-vale, CA). The band intensity was quantified using Image J 1.47 software and relative expression averaged across the three experiments.

### Statistical analysis

All variables were tested in three independent cultures for each experiment. Data are reported as the mean ± standard deviation (SD) and analyzed using one-way analysis of variance (ANOVA). All analyses were performed using SPSS 18.0 statistical software (SPSS, Inc, Chicago, IL, USA). P<0.05 was considered to indicate a statistically significant difference.

## Results

### Atorvastatin calcium inhibited PDGF-BB-induced proliferation of VSMCs

**Fig 1:**
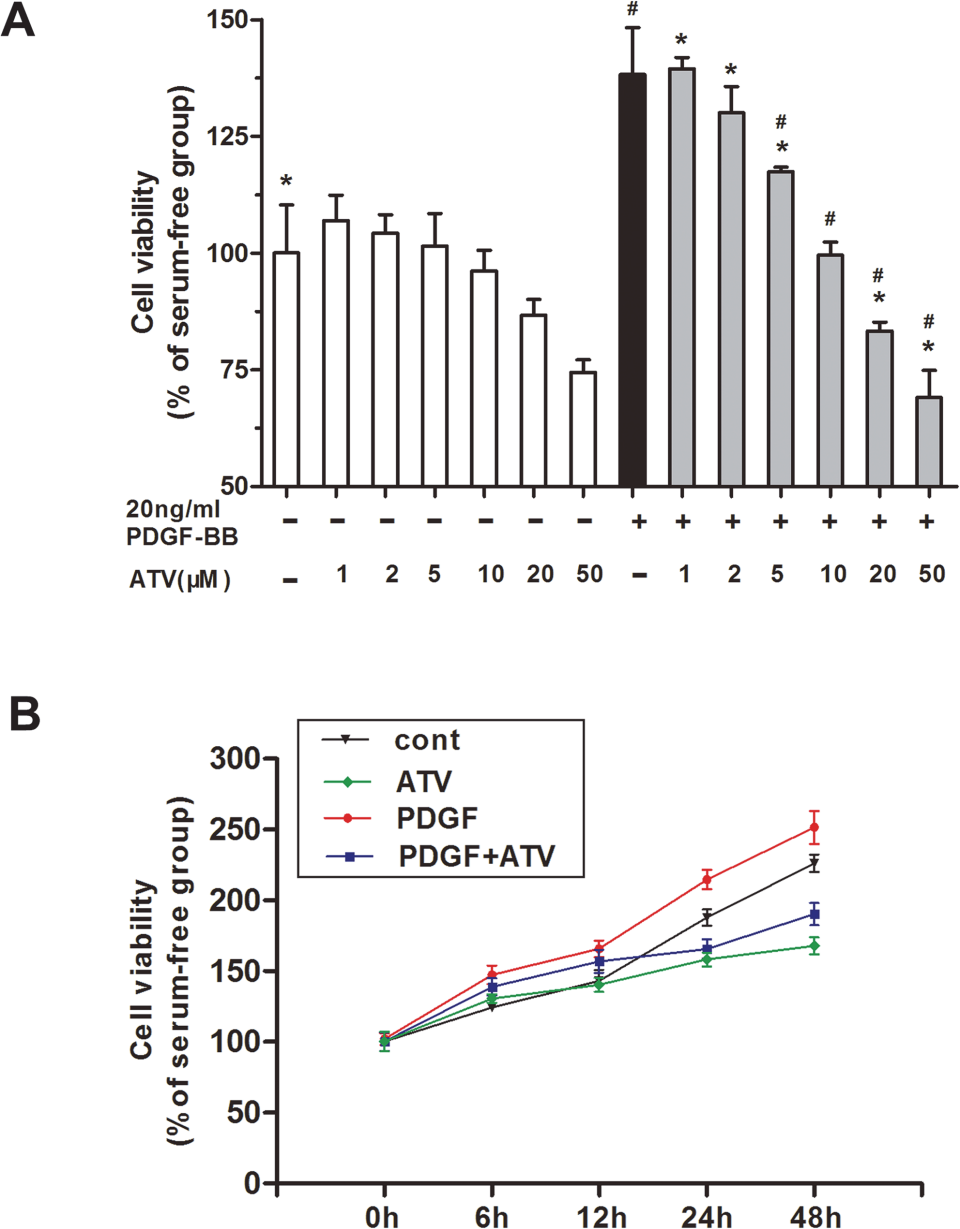
Antiproliferative activity of atorvastatin calcium (ATV) in PDGF-BB-stimulated VSMCs. After 24h of starvation with serum-free DMEM, VSMCs were incubated with 20ng/mL PDGF-BB or increasing concentrations (1–50μM) of atorvastatin calcium, alone or in combination for different hours (0-48h). (A) The inhibitory effect of increasing concentrations (1–50μM) of atorvastatin calcium on PDGF-BB-induced VSMCs. (B) The inhibitory effect of 10μM atorvastatin calcium on PDGF-BB-induced VSMCs for different hours (0-48h). *P < 0.05 compared with nonstimulated controls; #*P* < 0.05 compared with 20ng/mL PDGF-BB-induced controls.

The effect of atorvastatin calcium on the proliferation of PDGF-BB**-**induced VSMCs was studied using an MTT assay and EdU incorporation assay. After 24h of starvation with serum-free DMEM, VSMCs were incubated with 20ng/mL PDGF-BB and increasing concentrations of atorvastatin calcium, alone or in combination for different hours. As shown in Fig 1A, administering 1–10μM of atorvastatin calcium alone into VSMCs didn’t significantly inhibit cell survival, although it appeared to have significantly inhibitory effects at the concentrations of 20μM and 50μM. The results indicated that atorvastatin calcium had a significant inhibitory effect on PDGF-BB-induced VSMCs growth in a dose- and time-dependent manner compared to control group. Treatment with 20ng/mL PDGF-BB induced proliferation of VSMCs in comparison with nonstimulated cells. However, co-administrated with 1–50μM atorvastatin calcium for 24 hours resulted in significant (p<0.05) dose-dependent reduction in cell survival (Fig 1A). We found that the cell survival decreased distinctively (p < 0.05) with high dose of atorvastatin calcium (5μM, 10μM 20μM and 50μM). Furthermore, a time-dependent decrease in atorvastatin calcium-induced inhibitory effect on PDGF-BB-induced VSMCs proliferation was also observed at a fixed dose of 10μM (Fig 1B).

**Fig 2:**
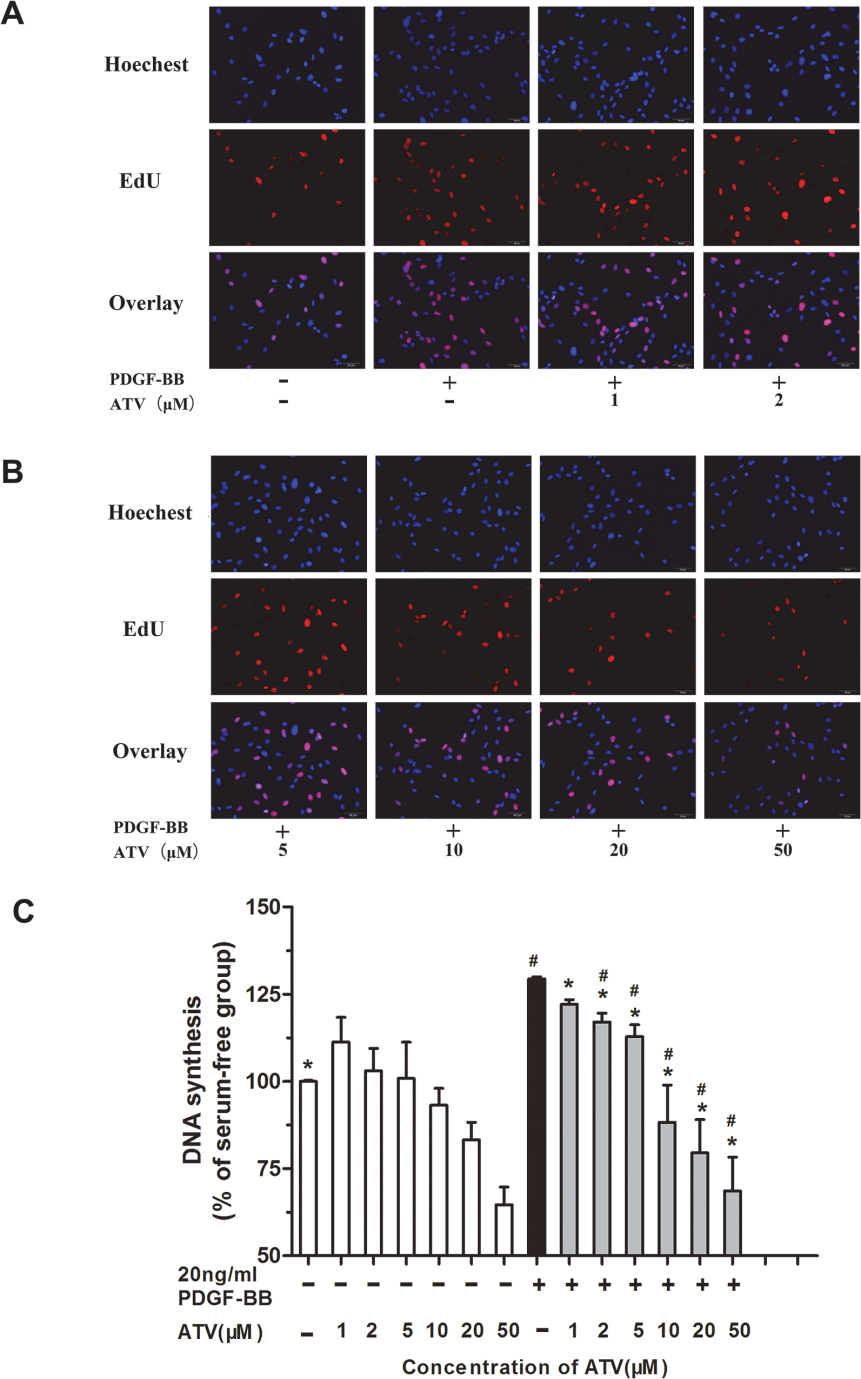
The effect of atorvastatin calcium (ATV) on PDGF-BB-induced DNA synthesis in VSMCs. DNA synthesis was detected using the EdU incorporation assay. (A)(B), EdU fluorescence staining to detect the newly synthesized DNA. (C) The percentage of EdU (+) cells was calculated by Image J 1.47 software.*P < 0.05 compared with nonstimulated controls; #*P* < 0.05 compared with 20ng/mL PDGF-BB-induced controls.

To investigate the mechanisms underlying altered cell growth, EdU incorporation assays were performed to examine the inhibitory effect of atorvastatin calcium on DNA synthesis. We found that 1–20μM of atorvastatin calcium alone did not inhibit EdU incorporation, but significant inhibition was observed at the concentration of 50μM. Stimulation of VSMCs with 20ng/mL PDGF-BB caused significant increase in the DNA synthesis, and atorvastatin calcium significantly (p<0.05) inhibited this increase in a concentration-dependent manner (Fig 2).

### PDGF-BB-induced VSMCs migration was inhibited by atorvastatin calcium

**Fig 3:**
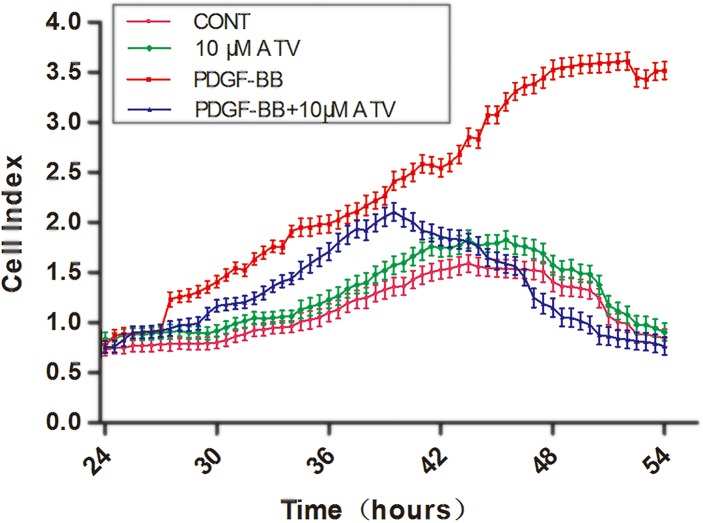
PDGF-BB-induced VSMCs migration was inhibited by atorvastatin calcium (ATV) in RTCA xCelligence migration assays. xCelligence was used to monitor real-time cell migration. VSMCs starved of serum for 24 hours were stimulated with 20ng/mL PDGF-BB only, 10μM atorvastatin calcium only, and PDGF-BB +ATV (CONT indicates that it contained only DMEM). Cell migration was then assessed by continuous resistance measurements for 30 hours. Experiments were performed in triplicate.

To determine the inhibitory effects of atorvastatin calcium on VSMCs migration, we performed RTCA xCelligence migration assays. The results showed that VSMCs migration was promoted by PDGF-BB and atorvastatin calcium (ATV) (10μM) did not increased cell migration when PDGF-BB was not present. However, the promotive effect of PDGF-BB-induced migration was conspicuously suppressed by the co-administration with atorvastatin calcium (Fig 3).

### Inhibitory Effect of atorvastatin calcium on PDGF-BB-induced VSMCs morphology and cytoskeleton rearrangement

To examine the phenotype modulation in PDGF-BB-stimulated VSMCs, we assessed VSMCs morphology and cytoskeleton using Rhodamine-phalloidin staining.

**Fig 4:**
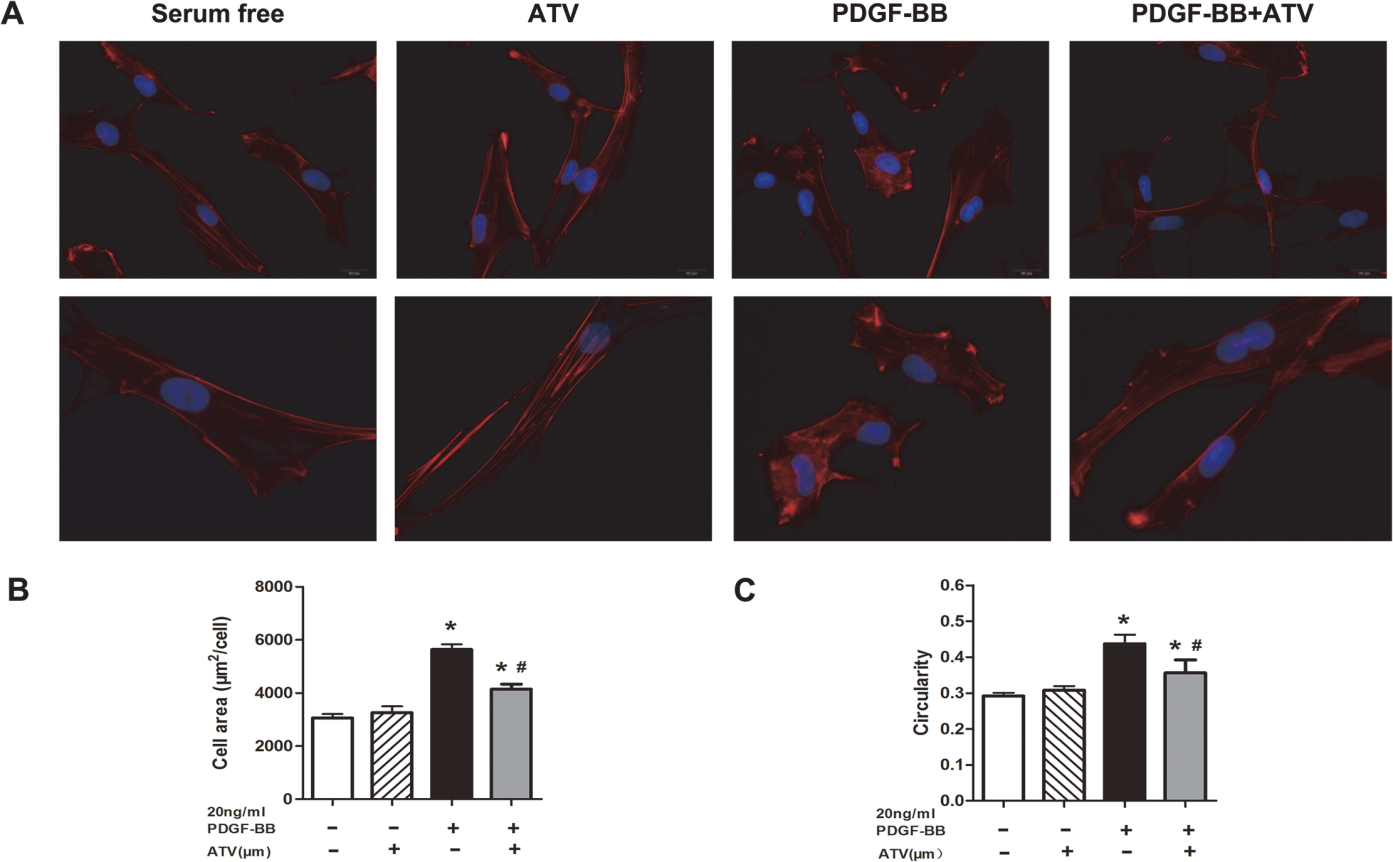
Characterization of morphology modulation and cytoskeleton rearrangement by atorvastatin calcium (ATV) on PDGF-BB-stimulated VSMCs. Cells were incubated in serum-free media, 20ng/mL PDGF-BB, or 10μM atorvastatin calcium, alone or in combination. Actin filaments were red due to Rhodamine-phalloidin, and nuclei were stained blue with DAPI. In (A), images in the upper and low panels were obtained at ×400 and ×1000 original magnifications, respectively. The micrographs shown in this figure are representative of three independent experiments with similar results. In (B), the average area (μm^2^/cell) ±SD was obtained from 100 single cells of each type. In (C), the average circularity±SD was obtained from 100 single cells per each type. *P < 0.05 compared with nonstimulated controls; #*P* < 0.05 compared with 20ng/mL PDGF-BB-induced controls.

As shown in Fig 4A, VSMCs for 24h starved exhibited elongation and spindle morphology in control group. It revealed an organized cytoskeleton network and aligned arrangement of actin filaments. Moreover,it did not show significant changes of cytoskeleton in VSMCs stimulated with atorvastatin calcium alone. In contrast, 20ng/ml PDGF-BB-stimulated VSMCs exhibited orderless distribution of actin filaments and aggregation around the perinuclear region without clear organized cytoskeleton network. However, VSMCs treated with atorvastatin calcium and PDGF-BB maintained spindle morphology and clear organization of the actin filaments by inhibiting the PDGF-BB-induced phenotype switching. Furthermore, after VSMCs were stimulated with PDGF-BB, cells exhibited morphological changes from spindle-shaped to polygonal shape, while co-treatment with atorvastatin calcium inhibited the morphological changes. As shown in Fig 4B, PDGF-BB-stimulated VSMCs had a greater area compared with serum-starved VSMCs. Atorvastatin calcium inhibited the change in area stimulated by PDGF-BB. Furthermore, the average circularity of PDGF-BB-induced cells was significantly higher than that of nonstimulated cells. However, VSMCs treated with atorvastatin calcium and PDGF-BB had lower circularity than the PDGF-BB group (Fig 4C).

### Atorvastatin calcium inhibited the PDGF-BB-induced reduction of VSMCs marker gene expression

**Fig 5:**
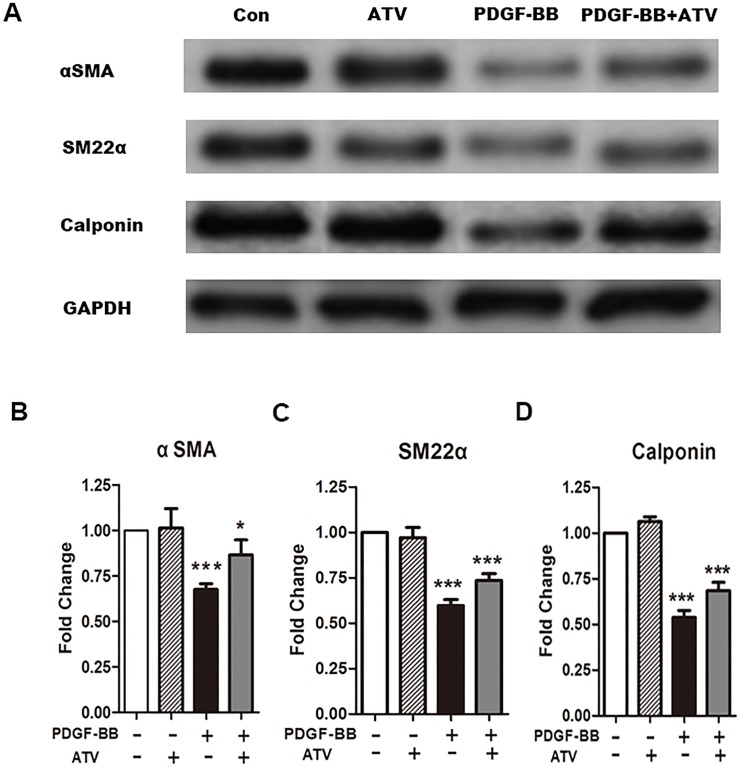
Atorvastatin calcium inhibited the PDGF-BB-induced phenotype switching of VSMCs. In (A), Con, VSMCs were cultured without any treatment; ATV, VSMCs were treated only with atorvastatin calcium (10μM) for 24h; PDGF-BB, VSMCs were treated only with PDGF-BB (20ng/ml) for 24h; PDGF-BB+ATV, VSMCs were treated with atorvastatin calcium (10μM) and PDGF-BB (20ng/ml) for 24h. The protein expression levels of the smooth muscle markers αSMA, SM22α and calponin were determined by western blotting. GAPDH, glyceraldehyde 3-phosphate dehydrogenase. In (B) (C) (D), the band intensity in (A) was quantified using Image J 1.47 software and relative expression averaged across the three experiments. Probability values are indicated above bars: *p<0.05, **p<0.01, ***p<0.001 versus control.

VSMCs are able to switch to a synthetic state in response to vascular injury. At the same time, they demonstrate low expression of SM-specific contractile markers, such as αSMA, SM22α and calponin. Therefore, we tested whether atorvastatin calcium can affect expression of VSMCs marker genes. After starvation, VSMCs were stimulated with PDGF-BB (20 ng/ml) and 10 μM atorvastatin calcium, alone or in combination for 24h. Western blotting data showed that the relative expression levels of αSMA, SM22α and calponin were markedly decreased in VSMCs stimulated with PDGF-BB (p<0.05) (Fig 5), indicating the dedifferentiation of the VSMCs into a synthetic phenotype. However, co-treatment with 10 μM atorvastatin calcium attenuated this effect, suggesting that atorvastatin calcium inhibited the PDGF-BB-induced reduction of VSMCs marker gene expression (p<0.05) (Fig 5).

### Inhibitory effect of atorvastatin calcium on the Akt signaling pathway activated by PDGF‑BB in VSMCs

**Fig 6:**
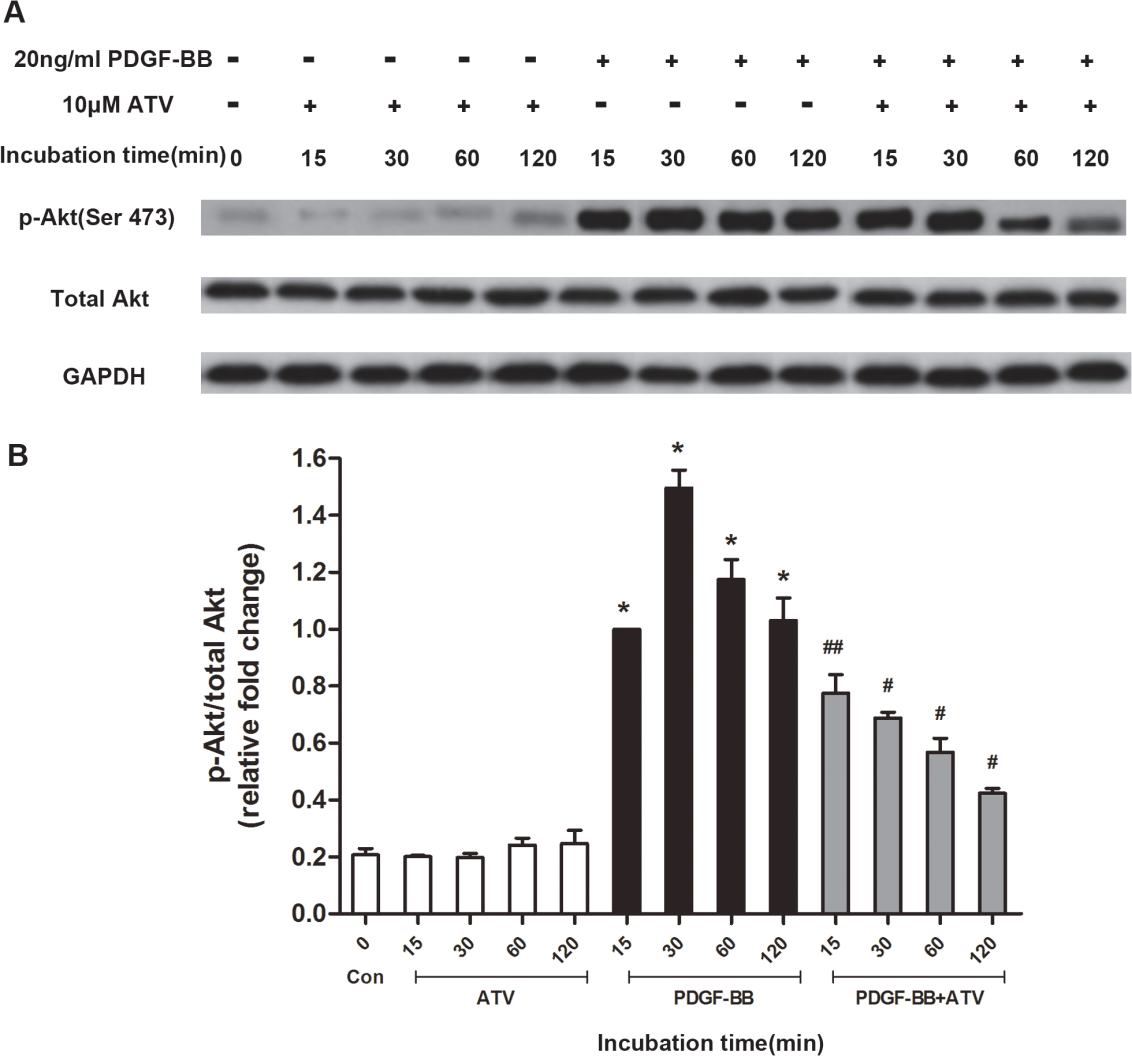
The effects of atorvastatin calcium(ATV) on modulation of PDGF-BB-induced signaling pathways in VSMCs. VSMCs starved of serum were stimulated with 20ng/mL PDGF-BB and 10μM atorvastatin calcium for the indicated times (15min, 30min, 1 h, and 2h) and lysed. Lysates were immunoblotted with antibodies. In (A), the time-dependent expressions of phospho-Akt. The band intensities were normalized to total Akt expression. In (B), the band intensity in (A) was quantified using Image J 1.47 software and relative expression averaged across the three experiments. Probability values are indicated above bars:*p<0.01,compared with nonstimulated controls (0min); #p<0.05, ##p<0.01, compared with 20ng/mL PDGF-BB-induced controls at different time.

It has been demonstrated that the Akt signaling pathway plays an important role in proliferation of PDGF-BB-stimulated VSMCs. Thus, we determined the activity of the Akt signaling pathway in VSMCs stimulated with PDGF-BB (20 ng/ml) with or without 10 μM atorvastatin calcium for specified times. As shown in Fig 6, atorvastatin calcium alone didn’t induce significant changes of Akt phosphorylation level, while the PDGF-BB stimulation elicited strong phosphorylation of Akt for several hours, atorvastatin calcium treatment significantly inhibited the PDGF-BB-mediated phosphorylation of Akt.

The western blotting data demonstrated that addition of 20ng/mL PDGF-BB to serum-starved VSMCs led to Akt phosphorylation that peaked within 30 min and lasting for at least 2 hours. However, 10 μM atorvastatin calcium treatment clearly inhibited the Akt phosphorylation induced by PDGF-BB in a time-dependent manner.

## Discussion

Proliferation and phenotypic modulation of VSMCs have been involved in the development and progression of cardiovascular diseases such as atherosclerosis, hypertension, and vascular injury[29, 31]. It has been demonstrated that vascular injury may affect VSMCs plasticity and lead to the dedifferentiation of VSMCs from a contractile phenotype into a proliferative phenotype[2, 3]. Identification of dedifferentiated VSMCs was based on morphological criteria that met the terms for “phenotypic modulation” or “phenotypic switching” in functional and structural properties.

PDGF-BB has been reported to participate in the pathogenesis of various vascular disorders. Here, we used PDGF-BB as a proliferative agent because previous study has demonstrated that PDGF is a key mediator in the proliferation of VSMCs[32]. It has already been reported that PDGF-BB is implicated in intracellular ROS generation and VSMCs growth[33, 34]. Furthermore, PDGF-BB inhibits the marker gene expression of VSMCs by activating several signaling pathways such as Akt pathway in cultured VSMCs[35]. Atorvastatin calcium is a selective HMG-CoA reductase inhibitor that has pleiotropic biological effects, which include inhibiting HMG-CoA reductase activity, increasing LDL receptor levels and inhibiting VLDL-C synthesis. As a result, atorvastatin calcium has been commonly used as an anti-hyperlipidemic therapy. Increasingly, evidence suggests that statins have effects beyond reduction of cholesterol levels. These pleiotropic effects highlight statins anti-inflammatory and immunomodulatory effects in cell and animal models[36]. In this study, we reported that PDGF-BB increased proliferation and migration, and decreased α-SMA, SM22α and calponin expression of VSMCs, indicating that VSMCs dedifferentiated into proliferative phenotype under stimulation with PDGF-BB. However, in our study, atorvastatin calcium inhibited the proliferation and migration of PDGF-BB-stimulated VSMCs. In contrast to the anti-proliferative effects of atorvastatin calcium on VSMCs, the pro-apoptotic properties of atorvastatin calcium had been clearly demonstrated in earlier studies. The authors found atorvastatin calcium only at the highest concentration tested (100μM) induced statistically significant levels of apoptosis[37]. In addition, we observed an increase in expression of contractile proteins and corresponding changes in cytoskeleton in VSMCs which were co-administrated with atorvastatin calcium and PDGF-BB. In other words, the contractile morphology and spindle phenotype of the PDGF-BB-induced VSMCs were preserved by atorvastatin calcium treatment. This study showed for the first time that atorvastatin calcium effectively suppressed PDGF-BB-induced VSMCs proliferation, migration and phenotypic modulation.

We further investigated the signal transduction pathways in PDGF-BB-stimulated VSMCs with or without atorvastatin calcium treatment. It has been reported that the Akt pathway regulates cell growth and cell differentiation in response to growth factors and cytokines[38, 39]. We demonstrated that the phosphorylation levels of Akt were significantly upregulated in PDGF‑BB‑stimulated VSMCs, while atorvastatin calcium co-treatment effectively attenuated the effect. This suggests that atorvastatin calcium might suppress phenotypic modulation of VSMCs induced by PDGF-BB via downregulation of the Akt pathway.

In this study, we focused on the inhibitory effect of atorvastatin calcium on PDGF-BB-induced phenotypic modulation of VSMCs. In summary, atorvastatin calcium exhibits various potential abilities to inhibit dedifferentiation of VSMCs, including anti-proliferative effect, decreasing the migration ability, maintaining the morphology and cytoskeleton of differentiated VSMCs, and an ability to modulate phenotypic switching, which are critical in neointimal hyperplasia, astherosclerosis and so on. We indicated that atorvastatin calcium showed promising effects for preventing the neointima formation associated with arteriosclerosis and restenosis subsequent to vein grafting or coronary intervention. Our study may enhance understanding of VSMCs phenotypic modulation and further provide potential therapeutic or preventive targets for cardiovascular diseases.

## Supporting Information

S1 Fig The purity of the isolated VSMCs. Immunofluorescence for smooth muscle α-actin in VSMCs (magnification, ×400).
(TIF)
