# Supplementary figures and images for: Atorvastatin Calcium Inhibits Phenotypic Modulation of PDGF-BB-Induced VSMCs via Down-Regulation the Akt Signaling Pathway
Source: PLoS One. 2015 Apr 15;10(4):e0122577. doi: 10.1371/journal.pone.0122577 (PMC4398430; doi:10.1371/journal.pone.0122577)

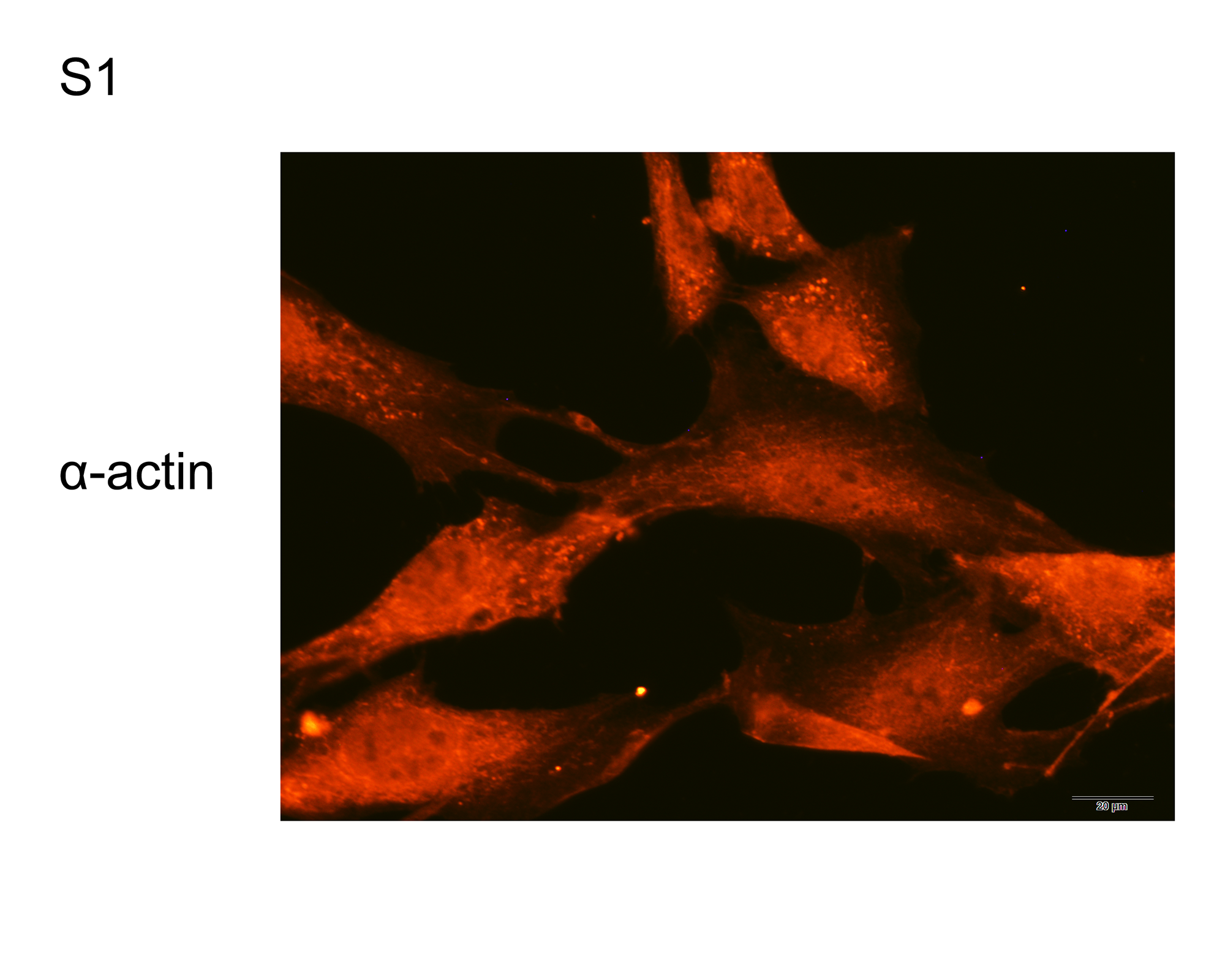

Supplement: S1 Fig — Immunofluorescence for smooth muscle α-actin in VSMCs (magnification, ×400). (TIF) [file pone.0122577.s001.tif]
